# Can governments promote homestead gardening at scale? Evidence from Ethiopia

**DOI:** 10.1016/j.gfs.2018.09.001

**Published:** 2018-12

**Authors:** Kalle Hirvonen, Derek Headey

**Affiliations:** International Food Policy Research Institute (IFPRI), 1201 Eye Street NW, Washington, DC 20005, USA

**Keywords:** AEW, Agricultural extension worker, BCC, Behavioral change communication, DGLV, Dark green leafy vegetables, EA, Enumeration Area, EHFP, Enhanced Homestead Food Production, HEW, Health extension worker, HG, Homestead garden, HKI, Helen Keller International, IYCF, Infant and young child feeding, NGOs, Non-governmental organizations, PSNP, Productive Safety Net Program, SNNP, Southern Nations, Nationalities, and Peoples' Region, WHO, World Health Organization, Homestead gardens, Adoption, Scalability, Water scarcity, Market access

## Abstract

Low intake of fruits and vegetables is a major cause of micronutrient deficiencies in the developing world. Since the 1980s, various non-governmental organizations have promoted homestead gardening (HG) programs, first in Asia, but now increasingly in Africa. Longstanding concerns with HG programs are: (1) they lack scalability, particularly for governments; (2) they only work in areas with/without good access to markets; and (3) they are only suitable for more water-abundant ecologies. We assess these concerns by analyzing a large and novel survey on the adoption of a nationwide HG program implemented by the Ethiopian government. We find that better market access encourages HG adoption; so too does greater public promotion of HGs, but only in more water-abundant ecologies.

## Highlights

• Homestead garden (HGs) programs are widely implemented in Africa.
• But no previous research analyses large-scale government programs.
• We use a rural survey covering households, extension workers, markets and climates in 264 rural Ethiopian communities.
• Adoption of HGs in water-scarce communities is low (<12%), suggesting water access is the main barrier to HG adoption.
• Encouragingly, though, government-led HG promotion does appear to be effective in more water-abundant communities.

## 1 Introduction

Malnutrition and poor diets rank first and second among level-2 risk factors for disability-adjusted life years in the 2015 Global Burden of Disease Study (Forouzanfar et al., 2015). One area where diets are widely deficient is in the consumption of fruits and vegetables (FVs), which are associated with increased risk of micronutrient deficiencies, heart disease, cancer, and obesity (Forouzanfar et al., 2015). As a result, nutritional guidelines recommend the consumption of at least two servings of fruits and three servings of vegetables per day, amounting to 400 mg (WHO/FAO, 2003). However, most people in lower income countries do not meet these requirements (Del Gobbo et al., 2015, Hall et al., 2009), largely because of affordability constraints. One recent study of FV prices in 18 low, middle and high-income countries estimated that low-income populations would need to spend half their daily income to meet recommended servings (Miller et al., 2016). A global study of 175 countries found that vegetables are especially expensive: dark green leafy vegetables (DGLVs) are 11–14 times as expensive a source of calories as staple cereals in sub-Saharan Africa, and 5–9 times as expensive in Asian regions (Headey and Alderman, 2017). In isolated rural areas, physical access to fruits and vegetables can be further limited by seasonality and lack of trade, especially for more perishable foods such as DGLVs.

Cognizance of the inadequately low intake of fruits and vegetables in rural areas of poor countries has long been recognized. In the 1980s, Helen Keller International (HKI) developed a homestead gardening (HG) program to increase FV consumption after a national blindness survey revealed that households with gardens were less likely to have children with night blindness. Over time, HKI's programs expanded and evolved into its *Enhanced Homestead Food Production* (EHFP) programs, which incorporate promotion of animal sourced foods (since the late 1990s), and an expanded set of behavioral change communication (BCC) components to improve child feeding and care practices (since the mid-2000s) (Haselow et al., 2016). HKI implemented EHFPs in a number of Asian countries, including Nepal, Cambodia, the Philippines, and more recently in Burkina Faso where a cluster-randomized control trial (cRCT) found significant and sizeable impacts on anemia, wasting and diarrhea in young children (Olney et al., 2015). And while HKI pioneered this approach, many other non-governmental organizations (NGOs) have adopted HGPs or EHFPs in different settings. In sub-Saharan Africa alone, NGO-led HG programs have been implemented in at least 22 countries.

Despite their popularity, reviews of these programs emphasize the lack of high quality experimental evidence of impact (i.e. randomized control trials), but also point to potential concerns over external validity and longer-term sustainability (Girard et al., 2012, Iannotti et al., 2009, Masset et al., 2012, Ruel et al., 2018, Webb and Kennedy, 2014). These are issues of significant concern for several reasons.

First, scalability and sustainability of HG programs is uncertain given that virtually all homestead food production programs have been implemented by NGOs. NGO staff are generally well trained and highly motivated to ensure the program is well implemented. It is therefore not certain that scaling up the program – or extending it to implementation by public officials – may have the same impacts (Deaton and Cartwright, 2018). Public health workers in developing countries often have multiple responsibilities that compete for their limited time and resources and are often subject to relatively weak monitoring and incentive mechanisms (Scott et al., 2018).

Second, the external validity of HG evaluations is questionable on ecological grounds. FVs are typically water-intensive crops, and reviews of HG programs have noted poor uptake in water constrained villages or dry seasons (Haselow et al., 2016). HKI's original FV programs were implemented in Asia, where rainfall levels are higher and small-scale irrigation much more widespread. In contrast, much of sub-Saharan Africa is water scarce, small-scale irrigation is rare (You et al., 2012), and much of the region is vulnerable to drier conditions due to climate change (Giannini et al., 2008). It is therefore unclear whether the lessons from programs implemented in areas characterized by good water access are directly exportable to water-scarce areas.

Finally, an outstanding concern among economists is whether improving homestead production is necessary for increasing consumption in places where markets might work reasonably well. Studies in Ethiopia, for example, find that farm production characteristics are strong predictors of diets, except when households have good access to markets (Hirvonen and Hoddinott, 2016, Hoddinott et al., 2015). Another recent study from Ethiopia shows that rural consumers obtain most of their non-staple foods from markets rather than from own production (Sibhatu and Qaim, 2017). This suggests that market access can substitute for household level production and allow farmers to specialize in producing a smaller set of foods more productively.

Despite these longstanding concerns, remarkably little research has rigorously assessed these issues. In this paper our primary objective is to assess the roles of public extension services, water availability and market access in influencing adoption of HGs in the important context of rural Ethiopia. Micronutrient deficiencies are widespread in Ethiopia, and in 2011 the average household consumed just 42 kg of F&V in a year per adult equivalent (Worku et al., 2017), far below the WHO recommendation of 146 kg per year (Hall et al., 2009). Unlike other contexts where NGOs largely implement HG programs in specific regions of a country (often more water-abundant regions), in Ethiopia the federal government is implementing a nationwide HG program. To our knowledge, this is the first study to evaluate HG adoption of a government-led program across such a diverse array of agro-ecological settings.

## 2 Methods

### 2.1 Context

This study focuses on the four most populous highland regions of Ethiopia; Amhara, Oromia, Southern Nations, Nationalities, and Peoples' Region (SNNP) and Tigray. These regions are largely covered by mountains and elevated plateaus that together cover about two thirds of the country and host more than 85% of the total population (CSA, 2013). Agricultural production is largely rain-fed and dominated by cereals and pulses (Bachewe et al., 2017). This study is further constrained to areas within these regions in which the Productive Safety Net Program (PSNP) operates. With 8 million beneficiaries, PSNP is one of the largest safety net programs in Africa. Geographically, the program is targeted to chronically food insecure districts (woredas). Most PSNP beneficiary households receive cash or food (mostly in the form of cereals) payments for undertaking public works while a small proportion of households with limited labour capacity receive unconditional payments.

Food security in these localities has been improving but remains high. The data underlying this paper show that the prevalence of stunting among children 6–23 months of age is high at 39%. Moreover, diets are extremely monotonous; the mean dietary diversity based on 24 h recall among children 6–23 months was 2.8 food groups (out of 7) and among mothers 2.2 food groups (out of 10). Less than 10% of the children (6–23 months) and mothers reported to have consumed Vitamin A rich fruits or vegetables in the past 24 h.

Homestead gardening has been a part and parcel of tropical food production for millennia (Kumar and Nair, 2004). This is also true in Ethiopia (McCann, 1995), although most small-scale “backyard” production still focuses on staple crops (enset, maize and teff) or stimulants (coffee, khat) (Mellisse et al., 2017), rather than nutrient-rich FVs. The Ethiopian government therefore seeks to promote small-scale FV production as an important tool to increase availability of FVs at the household and community level. Both the *National Nutrition Program* and the *Nutrition Sensitive Agriculture Strategy* set explicit targets for HG adoption: 40% of rural households by 2020, and 25% of urban households by 2020 (GFDRE, 2016, MoANR, & MoLF, 2016). The government's HG activities are implemented as part of a broader package of community-based nutrition-specific and nutrition-sensitive services, including BCC to promote age appropriate feeding practices, improved growth monitoring, treatment of severe acute malnutrition, disease prevention and management, social safety nets, and other agricultural interventions (FDRE, 2016, GFDRE, 2016). Many of the nutrition-specific activities are implemented by health extension workers (HEWs), who are also tasked with promoting household adoption of HGs. Community volunteers, known as the *Health Development Army*, assist HEWs in implementing these services, and agricultural extension workers (AEWs) are further tasked with providing technical support to households that wish to adopt. So far, HG activities have focused on promotion and technical support. The provision of inputs such as seeds and seedlings for HGs by the HEWs or AEWs has been rare. Moreover, the production of poultry or other small ruminants is not yet widely promoted either.

### 2.2 Data collection and measures

This study uses three types of data: (1) secondary household survey data collected by the authors in March and August in 2017 in PSNP districts in Amhara, Oromia, SNNP and Tigray; (2) community level data collected from HEWs, as well as from community food markets; and (3) Geographic Information Systems (GIS) data on agro-climatic factors.

### 2.3 Household survey data

The original purpose of these surveys was to obtain information for an evaluation of nutrition sensitive components of the PSNP. To this end, a stratified sample was drawn from areas in which the PSNP operates in the four highland regions. Given the focus of the original evaluation on outcomes related to child nutrition, the sample was restricted to poor households with a child less than 24 months of age in March 2017. Supplemental File S1 describes the sampling strategy in more detail.

**Fig. 1:**
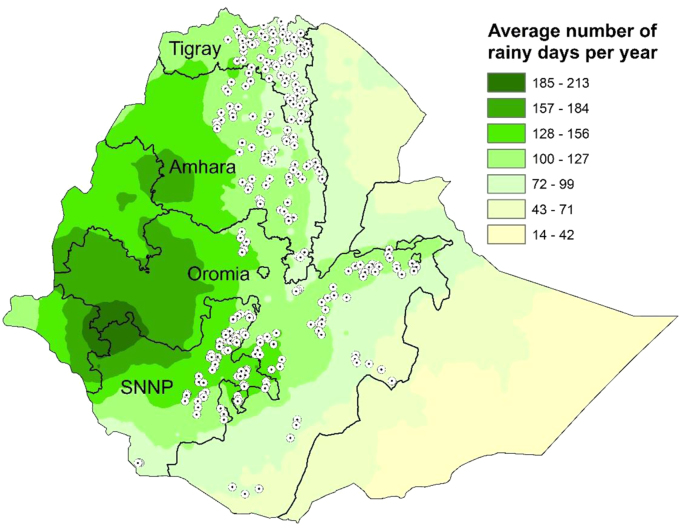
Locations of the 264 communities in the household survey and their overlap with average rainfall over 1997–2015.

While the geographic and demographic restriction is potentially a limitation on external validity, the survey has several useful characteristics. First, this survey is longitudinal and designed to capture seasonal differences. The first survey round was administered 3–4 months after the main harvest season in March 2017 (N = 2635), in what is typically a dry season. A second follow-up survey of the same households was conducted approximately six months later in August 2017 (N = 2569) during the long rainy season (2.5% of first round participants were not available in the August 2017 round). Second, the sample is large and geographically extensive (see Fig. 1), covering 264 enumeration areas (EA) from 264 sub-districts (*kebele*) and 88 districts (*woreda*). Moreover, although all 264 communities are poor, they vary substantially in terms of agro-climatic conditions, population density and access to markets. Lastly, the survey collected data on a wide range of household demographic and socioeconomic characteristics, including asset ownership and maternal nutrition knowledge (with the primary respondent being the mother of the young child).

### 2.4 Community level data

The field team also interviewed 249 HEWs working in the same localities in the August 2017 round, including questions on HGs. In addition, the nearest food markets of the communities (N = 264) were visited in both survey rounds. The market survey instrument recorded information on market characteristics (e.g. size of the market, infrastructure) and prices of 71 commonly consumed food items.

### 2.5 GIS data

For each sampled household and food market, we obtained latitude and longitude coordinates using Global Positioning System (GPS) devices. We used these data to compute the geodetic distance between the household and the nearest food market. We also linked the household level GIS data to daily weather data from National Aeronautics and Space Administration (NASA) and used these to compute the mean number of rainy days in the locality over 1997–2015. The location of the 264 communities across the four highland regions and their overlap with different rainfall brackets is depicted in Fig. 1.

### 2.6 Variables

We used these different data sources to construct an array of indicators of HG adoption and its determinants (details for all variables used in the analysis are provided in Supplemental File S2). HGs were defined as a small area next to the house in which the household cultivates FVs. In each survey round (March and August), households were asked whether they had cultivated a homestead garden in the past 12 months. Based on the responses to this question, we created a binary variable that obtained one if the household reported to have cultivated a HG in the past 12 months in either March or August. Respondents were also asked why they did not adopt a HG, with specific responses for “poor access to water”, “not enough available land”, “not enough time”, “not having seeds/inputs”, “not having the skills/knowledge”, along with a generic “other reasons” option. The survey also asked whether HEWs, volunteer health workers or AEWs promoted HGs. We used these responses to measure community level promotion intensity of HGs in each locality by taking the share of other households in the EA that reported receiving encouragement from extension workers (i.e. we excluded the household's own response when calculating the EA level promotion rate).

Two different measures of water access were used: 1) median reported time in the EA to fetch water (to go to the water source, get water, and come back) during dry season and 2) mean number of rainy days in the locality. Market access was measured using the quality of the nearest food market and household's distance to this market. To measure market quality, principal components analysis was used to construct an index from seven different market characteristics: Road accessibility; Road quality; Accessibility by public transportation (buses); Access to electricity; Access to cell phone network; Number of food traders; and Market type (permanent or temporary market). Further analysis (see Supplemental File S3) shows that physical accessibility of the market, size of the market and the availability of electricity are major determinants of this market quality index. The final market quality index used in the analysis varies in value between 0 and 10 with higher values indicating better market quality. Household's distance to the nearest food market is based on geodetic distance and constructed using GPS coordinates of the household and the market. Maternal nutrition knowledge was measured using mothers' responses to 12 questions regarding infant and young child feeding (IYCF) practices (see Supplemental File S4). The final maternal nutrition knowledge score used in the analysis varies in value between 0 and 10 with higher values indicating better knowledge.

### 2.7 Analyses

All statistical analyses were conducted using Stata, version 15.0 (StataCorp, Texas). The analysis proceeds in stages. We first use descriptive statistics to compare the characteristics of adopting and non-adopting households, along with two-sample *t*-tests of significant group differences. We then provide a regional bivariate analysis of households' homestead garden adoption and access to water and food markets. Multivariate probit methods were then used to test whether the observed associations between HG adoption and various household characteristics remained significant after controlling for observable confounding factors. We also ran a sensitivity analysis in which communities were split between water-abundant and water-scarce areas, as determined by mean rainfall levels over 1997–2015. In all regression analyses, the standard errors were clustered at the EA level.

## 3 Results

### 3.1 HG promotion and adoption rates and perceived barriers

Based on the data collected in August 2017, 86% of the HEWs reported promoting HGs to their clients in the past 12 months and 27% of households reported receiving encouragement to adopt HGs (Table 1) in the past 12 months. Supplemental Table S1 disaggregates the promotion rates by source and region as reported by the households in August. More than 20% of the households were encouraged to adopt a HG by a HEW, 19% by a AEW and 13% by a volunteer community health worker. Nearly all households that received encouragement from AEWs also reported receiving technical support on how to establish and operate a HG.

**Table 1:** Homestead garden promotion and adoption, by region.

*Source:*	*HEW in August (n = 249)*	*Household in August (n = 2569)*	*Household in March (n = 2635)*	*Household in August (n = 2569)*	*Household in March/August (n = 2569)*
	Promoted HG (%)	Received HG promotion (%)	Adopted HG (%)	Adopted HG (%)	Adopted HG (%)
Amhara	78.3	27.8	6.8	6.3	12.2
Oromia	76.7	8.9	4.0	5.0	8.3
SNNP	95.3	40.7	17.2	23.8	33.9
Tigray	90.8	30.1	4.7	3.2	7.5
**All**	**85.5**	**26.9**	**8.2**	**9.5**	**15.4**

Only 15% of the households (395 out of 2538 households) reported operating a HG in the past year (Table 1), either in March or August round. The adoption rates were substantially higher in SNNP (34%), which is considerably more rainfall-abundant than the other three regions. Adoption was lowest in Tigray (7.5%), a particularly water-scarce region.

Most gardens grew vegetables (Supplemental Table S2), with Ethiopian kale (a Vitamin A-rich leafy vegetable) the most popular crop (Supplemental Table S3). Most of the HG crop harvests (64%) took place between June and November (Supplemental Fig. S1) overlapping with the main rainy season (*kremt*), which typically extends from June to September. This suggests that the HGs in this context operate largely on a seasonal basis, rather than all year around.

Most non-adopters cited limited access to water (37%) and land (46%) as the main barriers to HG adoption (Table 2). Lack of skills/knowledge (7%), inputs (2%) and time (3%) were much less important barriers. There were, however, important regional differences in responses. For example, more than 50% of non-adopters in water-scarce Tigray reported poor access to water as the main constraint, while households in relatively more water-abundant but population-dense SNNP were more likely to cite land constraints (potentially this response could refer to lack of land of the requisite quality, rather than just sheer space).

**Table 2:** Reported constraints to homestead garden adoption among household that did not adopt, by region.

	**All (N = 2313)**	**Amhara (N = 592)**	**Oromia (N = 606)**	**SNNP (N = 485)**	**Tigray (N = 630)**
Water (%)	37.2	25.9	33.2	36.5	51.2
Land (%)	46.0	53.2	45.8	53.9	34.5
Time (%)	3.1	3.7	3.5	3.1	2.2
Skills/knowledge (%)	7.0	11.8	6.9	1.8	6.3
Inputs (%)	2.3	1.0	2.4	2.3	3.4
Other reason (%)	4.4	4.4	8.3	2.3	2.4
**Total (%)**	**100**	**100**	**100**	**100**	**100**

Note: Data pertain to households that had not adopted a homestead garden in the August round.

### 3.2 Factors associated with HG adoption

Table 3 compares the characteristics between non-adopters (n = 2143) and adopters (n = 395). Households with HGs are in localities with higher HG promotion rates (p < 0.001) compared to households that did not adopt a HG. HG adoption is also associated with better access to water; adopters have, on average, shorter distance to fetch water (p < 0.001) and more rainy days (p < 0.001) than non-adopters. Better market access is also positively associated with adoption: HG adopters are, on average, linked to better quality food markets (p < 0.01) and the mean distance to the nearest food market is marginally shorter (p < 0.05). Supplemental Table S4 further contrasts HG adoption rates to water, market and land access by region; water access and distance to markets are markedly better in SNNP, although market quality is rather low in SNNP as is the average land size. We also note that market access and water access are positively correlated (Supplemental Figs. S2 and S3).

**Table 3:** Sub-sample means for households with and without homestead gardens (N = 2538 ).

**Variable**	**without HG**	**with HG**	**difference**
	**n = 2143**	**n = 395**	
HG promotion intensity in the community	0.242	0.383	0.141***
Median distance to the water point (in hours) in the EA	0.700	0.583	− 0.117***
Average annual number of days with rainfall in the EA	179.1	211.7	32.6***
Household's geodetic distance to the nearest food market (in km)	6.511	5.910	− 0.601*
Market quality index (range: 0–10)	6.967	7.254	0.287**
Mother's age (in years)	28.72	29.96	1.240***
Mother's education (in years)	0.821	0.980	0.159
IYCF knowledge score (range: 0–10)	4.939	5.578	0.639***
Number of household members 0–5 years	1.718	1.866	0.148***
Number of household members 6–15 years	1.638	1.808	0.170*
Number of household members 16–60 years	2.275	2.357	0.082+
Number of household members 61+ years	0.0901	0.0987	0.009
Head is male	0.895	0.929	0.034*
Head age in years	37.96	38.29	0.330
Durable asset index (range: 0–10)	2.428	2.842	0.414***
Operated agricultural land area (in hectares)	0.841	0.763	− 0.078*
PSNP household	0.403	0.324	− 0.079*
Orthodox	0.503	0.367	− 0.136***
Muslim	0.321	0.329	0.008
Other religion	0.176	0.304	0.128***
Tigray region	0.279	0.124	− 0.155***
Amhara region	0.258	0.195	− 0.063**
Oromia region	0.270	0.134	− 0.136***
SNNP region	0.194	0.547	0.353***

Note: Equality in means between HG and non-HG households tested using a two-sample *t*-test.

*** p < 0.001.

** p < 0.01.

* p < 0.05.

+ p < 0.10.

As for maternal characteristics, mothers are older (p < 0.001) and their IYCF knowledge is better (p < 0.001) in HG-adopting households. Households with more children less than 5 years of age are also more likely to have adopted a HG (p < 0.001), possibly reflecting higher demand for FVs. We also see that the adopting households are wealthier as measured by the ownership of durable assets (p < 0.001) and less likely to be recipients of PSNP transfers (p < 0.05). In contrast to the qualitative evidence presented in Table 2, these bivariate associations do not suggest that access to land is an important constraint to adoption: HG households have less land, on average, than non-adopting households (p < 0.05).

Table 4 presents the marginal effects from a probit regression where the outcome variable obtains a value of one if the household adopted a HG in either survey round, and zero otherwise. HG promotion intensity in the community is associated with increased likelihood of adoption both in unadjusted (column 1) and adjusted models (column 2). Increasing the promotion rate in the EA by 10 percentage points is associated with a 1.1 percentage point increase in the likelihood that the household adopts a HG (p < 0.01), holding other factors constant. Considering the 15.4% adoption rate (Table 1), this translates into a 7% increase in the HG adoption likelihood. Increasing the distance to the water point by 30 min (0.5 h) is associated with a 4.2 percentage point (or 28%) decrease in the likelihood in adoption. Similarly, a 10% increase in the number of rainy days in the EA is associated with a 1.6 percentage point (or 10%) increase in HG adoption rate. The quality of the market is also positively associated with adoption (p < 0.01): increase in the market quality by 1 standard deviation (2.0 index units) is associated with a 3.4 percentage point (or 22%) increase in HG adoption rate. Finally, household's (geodetic) distance is also associated with adoption (p < 0.05). However, the coefficient on the logged variable is close to zero indicating that the magnitude of the association is weak.

**Table 4:** Determinants of homestead garden adoption – marginal effects from probit regressions.

	**1**	**2**
	**unadjusted**	**adjusted**
	n = 2538	n = 2538
HG promotion intensity in the community	0.199***	0.107**
median distance to the water point (in hours) in EA	− 0.081***	− 0.085**
(ln) average annual number of days with rainfall in EA	0.380***	0.154*
(ln) distance to the nearest weekly food market	0.012	0.018*
market quality index	0.016*	0.017**
mother's age (in years)		0.004**
mother's education (in years)		0.005
nutrition knowledge score		0.007*
number of household members 0–5 years		0.031**
number of household members 6–15 years		0.001
number of household members 16–60 years		0.003
number of household members 61+ years		0.033
head is male		0.047+
head is female		reference
head age in years		− 0.001
wealth index		0.021**
number of tropical livestock units (TLU) owned		0.003
(ln) land area (in hectares) operated		− 0.007
PSNP household		− 0.011
Orthodox		0.148***
Muslim		0.104**
other religion		reference
Amhara region		0.075**
Oromia region		0.086*
SNNP region		0.256***
Tigray region		reference

Note:

Standard errors were constructed using the delta method and clustered at the EA level.

*** p < 0.001.

** p < 0.01.

* p < 0.05.

+ p < 0.10.

The foregoing results suggest that access to water is the most important determinant of HG adoption. We therefore hypothesized that the association between HG adoption and promotion intensity varies across the water access dimension: that is, efforts to promote HG adoption in water-constrained areas is akin to pushing on a string. To test this, we re-ran the unadjusted and adjusted models presented in Table 4 for sub-samples in which the number of rainy days is below the sample median (172 days of rain per annum) and areas in which the annual number of 229 rainy days is above the median. The results, presented in Table 5, suggest that HG promotion is not effective in water-scarce areas; the estimated coefficient is nearly zero and not statistically significant (p = 0.973; adjusted model). In contrast, HG adoption is strongly associated with HG promotion in water-abundant areas (p = 0.001; adjusted model). The coefficients on the other key variables are similar across the two samples, except for distance to the nearest water collection point. In the adjusted models, the negative coefficient on this indicator is substantially larger in more water-abundant areas. We hypothesize that even in rainy areas households still use collected water to irrigate their FV plots, whereas severe rainfall constraints preclude FV production in the first place in water-scarce areas, making water collection irrelevant.

**Table 5:** The relationship between homestead garden adoption and promotion in water-scarce and water-abundant sub-samples – marginal effects from probit regressions.

	**1**	**2**	**3**	**4**
	**Water-scarce communities (EAs)**		**Water abundant communities (EAs)**	
	**unadjusted**	**adjusted**	**unadjusted**	**adjusted**
	n = 1206	n = 1206	n = 1332	n = 1332
HG promotion intensity in the community	0.021	0.001	0.377***	0.163**
median distance to the water point (in hours) in the kebele	− 0.081**	− 0.005	− 0.115**	− 0.137***
(ln) average annual number of days with rainfall in EA	omitted	omitted	omitted	omitted
(ln) distance to the nearest weekly food market	− 0.005	0.001	0.021	0.032*
market quality index	0.015*	0.018*	0.018+	0.020*
mother's age (in years)		0.002		0.007**
mother's education (in years)		0.002		0.009
nutrition knowledge score		0.006		0.007
number of household members 0–5 years		0.019+		0.036*
number of household members 6–15 years		0.002		− 0.001
number of household members 16–60 years		0.012		− 0.002
number of household members 61+ years		0.036		0.038
head is male		0.049		0.040
head is female		reference		reference
head age in years		− 0.001		− 0.003*
wealth index		0.013+		0.020+
number of tropical livestock units (TLU) owned		− 0.009*		0.014**
(ln) land area (in hectares) operated		0.004		− 0.017*
PSNP household		− 0.032*		− 0.001
Orthodox		0.034		0.142**
Muslim		− 0.036		0.121**
other religion		reference		reference
Amhara region		0.057**		0.157**
Oromia region		− 0.010		0.195**
SNNP region		0.115*		0.401***
Tigray region		reference		reference

Note: .

Standard errors were constructed using the delta method and clustered at the EA level. Water-scarce = ‘average annual number of days with rainfall in EA’ is below median. Water-abundant = ‘average annual number of days with rainfall in EA’ is above median. The sample mean for 'share of other EA households for which HG promoted' is 0.27 in 'water scarce' sub-sample and 0.25 in 'water abundant' sub-sample.

*** p < 0.001.

** p < 0.01.

* p < 0.05.

+ p < 0.10.

**Fig. 2:**
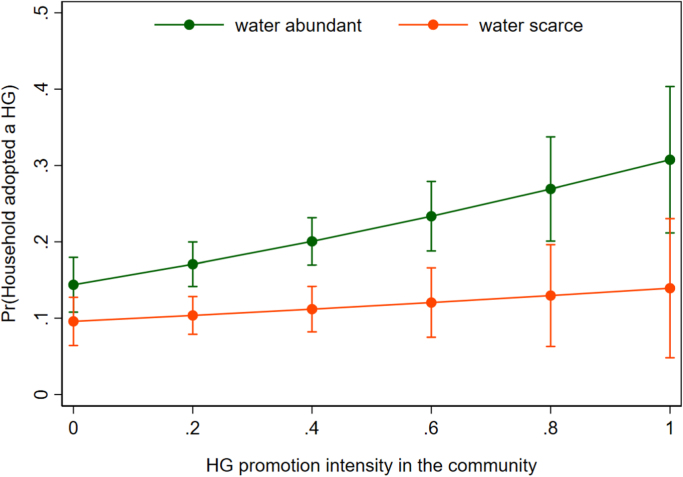
Predictive margins for different levels of HG promotion intensity by community's water access. Note: Predictive margins for different levels of HG promotion intensity. The vertical capped lines show the 95% confidence intervals for the point estimates. 90% of the promotion intensity observations lie between 0.0 and 0.7.

Next, we provide a more formal test of the differences in association between HG adoption and promotion intensity in water-scarce and water-abundant localities. Instead of splitting the sample, we appended the adjusted model with a term capturing the interaction between water-scarcity and promotion intensity. Fig. 2 shows the predictive margins derived from this probit regression. As before, we see that the HG promotion intensity is associated with increased adoption only in water-abundant areas. The difference in the point estimates is statistically significant at 95% level for most of the HG promotion intensity distribution, providing further evidence that water constraints render HG promotion ineffective.

## 4 Discussion

To our knowledge this is the first study to extensively examine adoption of a government-led HG program implemented across a diverse set of agro-ecologies. Qualitative evidence suggests that land and water are the two most important constraints, but quantitative evidence emphatically points to water as the more binding constraint in most communities. Water availability would also appear to explain why the SNNP region has much higher adoption rates than the other three highland regions, where fewer than 10% of households adopted HGs. The fact that more households in water-abundant SNNP cite land as a constraint leads us to hypothesize that land/soil constraints only become binding once water is no longer a binding constraint.

More encouragingly, we find that community level promotion of HGs by extension workers is strongly and positively associated with adoption, though only in sufficiently water-abundant communities. This suggests that, in the Ethiopian context at least, the public extension system may be an effective institution for changing households’ production choices where ecological constraints are not binding. Also of relevance, especially from an economic viewpoint, is that market quality is positively associated with HG adoption, suggesting that having a marketing outlet for FVs encourages adoption. In retrospect, such a result is perhaps not surprising, since these are very poor cash-constrained communities. However, the role of markets in encouraging adoption does suggest that further research is needed on understanding the impacts of HG adoption on household and child-level FV consumption, household incomes, and community-level availability/affordability of FVs.

This study has limitations. First, external validity is a potential concern given the survey's restriction to chronically food insecure areas. That said, this concern may not be important in practice, since most of rural Ethiopia is poor in absolute terms, such that we expect a national level survey to yield broadly similar results. It is less certain that our results on government-led HG promotion are likely to extend to other countries given the very context-specific institutional dimensions of extension services. For one thing, Ethiopia's public extension system is larger (more agents per farmer) and perhaps more effective than other sub-Saharan African countries' (Berhane et al., 2018, Davis et al., 2010). Second, qualitative data from household and HEW interviews may be subject to response biases. Indeed, although many households cited land availability as a constraint, we found little empirical support for that claim – although it may be that in their responses households were referring to specific land qualities that are favorable for FV production (e.g. soil quality). HEWs may also be more likely to over-report HG promotion activities to give the impression that they are fulfilling their socially mandated responsibilities, although we did find significant associations between HEW reports and household reports of exposure to extension services. Third, our measure of HG adoption is a simple binary variable that does not capture quantities of FVs produced or nutrient availability. Lastly, our analysis is observational: we explore potential determinants of adoption that can only be assessed through the lens of plausibility rather than strict causality.

Bearing these caveats in mind, our results have important implications for HG programs in Ethiopia, and perhaps elsewhere in sub-Saharan Africa. The data suggest that Ethiopia's health and agricultural extension workers have been active in promoting HGs, and that these efforts may well lead to greater adoption, although only in more water-abundant ecologies. While previous studies suggest that extension workers in Ethiopia are often overburdened, they also find that they have been an effective instrument for promoting a wide variety of health and sanitation programs (Berhane et al., 2018, Davis et al., 2010, Mangham-Jefferies et al., 2014, Tilahun et al., 2017, Workie and Ramana, 2013). While these results may be very specific to Ethiopia's relatively large public health and agricultural extension systems, it is encouraging to see indicative evidence that public extension workers may be successful in changing agricultural practices in appropriate agro-ecological settings, since prior evidence on HG promotion/adoption from other countries was invariably restricted to NGO workers.

On a less positive note, the clear importance of water availability for FV production calls for more strategic thinking about the viability and cost-effectiveness of promoting HGs in water-constrained communities, which describes a large part of the Ethiopian highlands and many other localities in Africa. In water-scarce ecologies sequencing of programs needs to be given more thought: investments to improve water access through drip irrigation or other small-scale infrastructure measures must precede FV programs. Ethiopia has made investments in irrigation, though mostly in large-scale irrigation schemes, and with mixed success (Awulachew, 2010, Berhane, 2018, Berhane et al., 2016). So far there has been little progress in decreasing travel times to water points to reduce women's workloads in rural Ethiopia (Hirvonen, 2017), although Ethiopia's Productive Safety Net Program is taking steps to address this issue in communities in which it operates in (Bossuyt (2017). Outside of Ethiopia, HKI programs have long recognized water scarcity as a barrier to HG adoption (Haselow et al., 2016, Olney et al., 2016, Olney et al., 2015), particularly in translating their programs from Asian to African contexts. This suggests that HG promotion efforts in sub-Saharan Africa need to be much more geographically selective, or combine water investments with HG promotion.

Finally, our results justify more research and policy consideration of markets and their role in providing nutritious foods at different times of the year. HG adoption was strongly associated with our index of market quality while the corresponding association with the more commonly used metric of “market access”, distance to the market, was weak. This should prompt researchers to think more carefully about what is meant by market access, and what makes a market high quality from a nutritional perspective. Indeed, surprisingly little is known about how effective rural markets are at providing affordable access to nutrient-rich foods in developing countries, whereas there is an extensive literature focusing on farm production and consumption of nutrient-rich foods (Sibhatu and Qaim, 2018).

One interesting line for future research is to understand how the importance of markets influences the nutritional impacts of HG promotion. HG programs have long assumed that FV production increases will mostly be consumed by beneficiary households, or that market sales will improve the diets of those households via income effects. However, it is also possible that HG adoption at scale may improve the affordability and accessibility of FVs in local markets, potentially to the benefit of “non-beneficiary” households. To our knowledge, however, no previous research on homestead production has examined spillovers on non-beneficiaries, even though widespread HG adoption could clearly alter local FV prices.

## Conflict of interest

Kalle Hirvonen – no conflicts of interest;

Derek Headey – no conflicts of interest.
