# Supplementary material for: Can governments promote homestead gardening at scale? Evidence from Ethiopia
Source: Glob Food Sec. 2018 Dec;19:40–7. doi: 10.1016/j.gfs.2018.09.001 (PMC6333280; doi:10.1016/j.gfs.2018.09.001)
Supplement: Supplementary file 1 — Supplementary material [file mmc1.docx]

**Can governments promote homestead gardening at scale? Evidence from Ethiopia**

**Supplementary material**

## Supplemental File S1: Sampling

The sampling for this survey was done in stages. First, 88 woredas were randomly selected from the full list of PSNP woredas in Amhara, Oromia, SNNP and Tigray regions. Three kebeles were randomly selected from each woreda. The Central Statistical Agency (CSA) of Ethiopia divides each kebele into enumeration areas (EA) that are roughly of equal size, typically containing about 200 households. From each kebele, using the list of EAs provided to us by the CSA, we randomly selected one EA from each kebele. The final step in the sampling procedure was to identify all households in the EA that had a child less than 24 months of age present in the household. If a young child was found residing in the household, the enumerators carefully probed his/her age to make sure that the child was indeed less than 24 months of age. A total of 46,866 households were listed residing in the EAs selected for this study. Out of these, 10,318 households reported that they had a child less than 24 month of old.

Once the EA level census was completed, we randomly selected 10 eligible households from each EA to be part of the survey. A household was eligible if it had a child less than 24-month-old. We further stratified the sample so that roughly half of the selected households were PSNP beneficiaries and half of them are poor but not benefitting from the program. Given the focus on poor households, this sample is not representative of the EAs or kebeles in which the sample was drawn. In total, 2,635 households with children less than 24 months were visited in 88 woredas, 264 kebeles in Amhara, Oromia, SNNP and Tigray.

The original purpose of the survey was to serve as a baseline to the evaluation of the nutrition sensitive components of the PSNP. Reflecting this. the sample size and the number of clusters were based on statistical power calculations and assumptions about attrition that permit the evaluation team to detect a 10 percentage point increase in child’s consumption of dairy products, a 12 percentage point increase in the likelihood that a mother has at least four antenatal care visits and a 10 percent increase in women’s body-mass index.

Finally, the survey team also collected price and market characteristics data from all community food markets. In addition, the survey team also visited the health post and interviewed one health extension worker working in the kebele. Most health posts in rural Ethiopia have two HEWs. Out of these, the enumerators interviewed the HEW that was available and willing to participate. A HEW interview was successfully conducted in 221 out of the 264 kebeles. In 43 kebeles, the HEW was not present either because the kebele did not have a health post or because none of the HEWs were present at time of interview.

## Supplemental File S2: Variable construction

| **Variable** | **Note on construction** |
| --- | --- |
| Household adopted a HG | In each survey round (March and August), households were asked whether they had cultivated a homestead garden in the past 12 months. Based on these responses, we created an indicator variable that obtained one if the household reported to have cultivated a HG in the past 12 months either in March or August. |
| HG promotion intensity in the community | Calculate the total number of households in the EA that reported to have ever received HG promotion and divide this with the total number of households in the EA. This is a *non-self-mean;* household's own response is not considered in the calculation. |
| Median distance to the water point (in hours) in the EA | In each survey round households were asked to estimate the time in minutes in takes for them to fetch water (i.e. go to the water point, get water and come back) during the rainy and dry seasons. We used the responses about the dry season when access to water is more constrained. In case of different responses in March and August, we first took the mean time reported by the household. After that, we calculated the median time reported by the households in the same EA. |
| Average annual number of days with rainfall in the EA | This variable was calculated using global precipitation data provided by the NASA and linked to the household data using GPS coordinates. Mean number of days with rainfall was calculated using daily rainfall observations for the EA over 1997-2015. |
| Household's geodetic distance to the nearest food market (in km) | The survey team collected GPS coordinates of the households and the nearest food markets. Using these coordinates, a geodetic distance between the household and the nearest food market was calculated. |
| Market quality index (range: 0-10) | See Supplemental File S1; market characteristics measured in the August round were used. |
| Mother's age (in years) | See Supplemental File S2; mother's responses in the August round were used. |
| Mother's education (in years) | Mother's responses in the August round were used. |
| IYCF knowledge score (range: 0-10) | Mother's responses in the August round were used. |
| Number of household members 0-5 years | Household's responses in the August round were used. |
| Number of household members 6-15 years | Household's responses in the August round were used. |
| Number of household members 16-60 years | Household's responses in the August round were used. |
| Number of household members 61+ years | Household's responses in the August round were used. |
| Head is male | Household's responses in the August round were used. |
| Head age in years | Household's responses in the August round were used. |
| Durable asset index (range: 0-10) | Household's responses in the August round were used. |
| Operated agricultural land area (in hectares) | Household's responses in the August round were used. |
| PSNP household | Household's responses in the August round were used. |
| Household religion | Household's responses in the August round were used. |

## Supplemental File S3: Description of the Construction of the Market quality index

The market survey instrument provides rich information about the community food markets:

1. Number of food traders
2. Type of market: permanent, semi-permanent or temporary market
3. Road quality
4. Road accessibility
5. Access with public transportation (buses)
6. Electricity
7. Cell phone coverage

We reduced these market characteristics into one 'market quality' index using principal components analysis. The seven variables are highly correlated (average correlation coefficient is 0.257) and the principal components analysis attempts to find components that account for most of the variation among these characteristics. The end-product is a single variable that we took to represent market quality.

Table below shows the eigenvalues for each of the seven components. We see that the first two components explain 55 percent of the variation in the data. We then followed the Kaiser-rule that states that only components that obtain an eigenvalue larger than one should be retained.

**Principal components and eigenvalues**

|  | **Eigenvalue** | **Proportion** |
| --- | --- | --- |
| component 1 | 2.717 | 0.39 |
| component 2 | 1.157 | 0.17 |
| component 3 | 0.996 | 0.14 |
| component 4 | 0.846 | 0.12 |
| component 5 | 0.492 | 0.07 |
| component 6 | 0.482 | 0.07 |
| component 7 | 0.310 | 0.04 |

Columns 1 to 2 in table below provide the principal component loadings based on the first three components. The Kaiser-Meyer-Olkin (KMO) measure of sampling adequacy is displayed in column 3. We see that most KMO values are close to one, suggesting that the 7 market characteristics are indeed measuring a common component.

**Principal component loadings and sampling adequacy**

|  | **1** | **2** | **3** |
| --- | --- | --- | --- |
| **Market characteristic** | **Comp 1** | **Comp 2** | **Kaiser-Meyer-Olkin measure** |
| Number of food traders | 0.43 | -0.33 | 0.77 |
| Market is permanent or semi-permanent | 0.18 | 0.42 | 0.53 |
| Good road quality | 0.26 | 0.68 | 0.63 |
| Good road accessibility | 0.43 | 0.31 | 0.74 |
| Accessible with public transportation (buses) | 0.48 | -0.27 | 0.75 |
| Access to electricity | 0.49 | -0.27 | 0.69 |
| Good cell phone coverage | 0.24 | 0.08 | 0.77 |
| **Overall** |  |  | 0.72 |

Finally, the market quality index based on the principal components analysis was scaled so that it obtains values between 0 and 10.

## Supplemental File S4: Description of the Construction of the Maternal Nutrition Knowledge Score

The nutrition knowledge module in the household questionnaire tested mothers' knowledge about complementary feeding through a battery of questions. The responses to the 12 questions were marked and the correct response to each question received one point. Table below displays the questions and the percent of mothers who answered correctly. In contrast to knowledge about breastfeeding related issues (see above), we observe poor knowledge regarding complementary feeding related issues (e.g. sources of important nutrients, consequences of nutrient deficiencies). Overall, these findings resonate with earlier research on maternal nutrition knowledge in Ethiopia (Abebe, Haki, and Baye 2016, Kim et al. 2015). The table further shows marginal differences between PSNP and non-PSNP households as well across survey rounds.

Correct responses to questions about IYCF practices in August round

|  |  | **%** |
| --- | --- | --- |
| 1 | Consequences of iron deficiency * | 48 |
| 2 | Sources of iron * | 34 |
| 3 | Consequences of vitamin A deficiency * | 39 |
| 4 | Sources of vitamin A * | 25 |
| 5 | What seasoning is often fortified with iodine | 61 |
| 6 | Are gruels traditionally prepared too thin | 17 |
| 7 | Food types to complement breastfeeding * | 67 |
| 8 | Can a 1-year old child eat alone without any supervision of an adult | 88 |
| 9 | Minimum meal frequency | 80 |
| 10 | What should a mother do when her child older than 6m has diarrhea * | 75 |
| 11 | How MUCH should a child be fed when s/he is sick | 33 |
| 12 | How OFTEN should a child be fed when s/he is sick | 37 |

* = more than one correct response; a point given if the respondent identified at least one of them.

We aggregated these responses into score. Each correct response was given one point, yielding an IYFC score ranging between 0 and 12.

Finally, this nutrition knowledge score was scaled so that it obtains values between 0 and 10.

**Table S1: Percentage of households for which homestead garden was promoted, by source and by region**

|  | **N** | **Any source** | **HEW** | **AEW** | **HDA** | **Technical support from AEW** |
| --- | --- | --- | --- | --- | --- | --- |
| Amhara | 632 | 27.8 | 21.4 | 19.3 | 11.9 | 16.8 |
| Oromia | 638 | 8.9 | 6.9 | 4.2 | 1.1 | 3.4 |
| SNNP | 636 | 40.7 | 29.6 | 27.8 | 18.9 | 26.3 |
| Tigray | 651 | 30.1 | 23.8 | 22.7 | 20.4 | 19.8 |
| **All** | **2,557** | **26.9** | **20.4** | **18.5** | **13.1** | **16.6** |

Note: As reported by the households. Source: August round. HEW = Health Extension Worker, AEW = agricultural extension workers, HDA = Health Development Army (volunteer health worker)

**Table S2: Food groups grown in homestead garden, reported by households**

| **Food group** | **N** | **%** |
| --- | --- | --- |
| Vitamin A-rich dark green leafy vegetables | 595 | 66.7 |
| Other vitamin A-rich vegetables and fruits | 42 | 4.7 |
| Other vegetables | 233 | 26.1 |
| Other fruits | 22 | 2.5 |
| **Total** | **892** | **100** |

**Table S3: Crops grown in homestead gardens, reported by households**

| **Crop** | **N** | **%** |
| --- | --- | --- |
| Ethiopian Kale | 553 | 62.0 |
| Onions | 68 | 7.6 |
| Green Pepper | 54 | 6.1 |
| Garlic | 37 | 4.2 |
| Spinach | 35 | 3.9 |
| Pumpkin | 25 | 2.8 |
| Tomato | 23 | 2.6 |
| Carrot | 14 | 1.6 |
| Cabbage | 14 | 1.6 |
| Beet Root | 12 | 1.4 |
| Lettuce | 10 | 1.1 |
| Bananas | 9 | 1.0 |
| Papaya | 8 | 0.9 |
| Avocado | 7 | 0.8 |
| Mango | 7 | 0.8 |
| Spices | 5 | 0.6 |
| Orange | 3 | 0.3 |
| Sweet Potato | 2 | 0.2 |
| Gisheta (custard apple) | 2 | 0.2 |
| Guava | 2 | 0.2 |
| Ginger | 1 | 0.1 |
| Apple | 1 | 0.1 |
| **Total** | **892** | **100** |

**Figure S1: Harvest times of homestead garden crops**


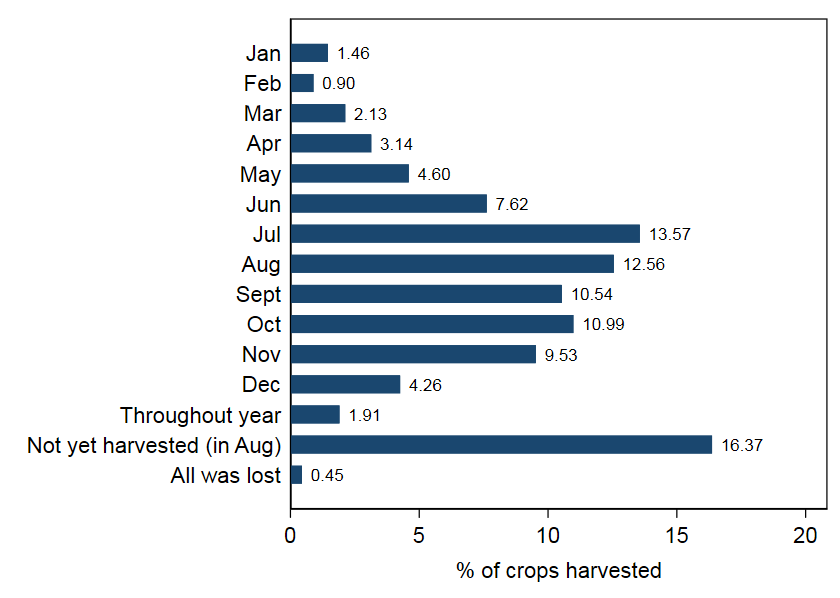


Note: The main rainy season (kremt/meher) takes place between June and September. Households that operated a HG but had not yet harvested in August when we visited them are reported under 'Not yet harvested (in Aug)'.

**Table S4: Homestead garden adoption rate, water access, market access and land size, by region**

|  | **N** | **Operates HG (%)** | **Mean travel time to fetch water**  **(in hours)** | **Mean number of rainy days in a year** | **Mean market quality index** | **Mean distance to market**  **(in km)** | **Median land size**  **(in hectares)** |
| --- | --- | --- | --- | --- | --- | --- | --- |
| Amhara | 632 | 12.2% | 0.51 | 173.9 | 6.8 | 6.7 | 0.83 |
| Oromia | 638 | 8.3% | 1.09 | 177.7 | 7.0 | 7.3 | 0.55 |
| SNNP | 637 | 33.9% | 0.64 | 238.9 | 6.4 | 4.0 | 0.50 |
| Tigray | 651 | 7.5% | 0.49 | 147.1 | 7.7 | 7.6 | 0.73 |
| **All** | **2,558** | **15.4%** | **0.68** | **184.2** | **7.0** | **6.4** | **0.63** |

**Figure S2: Relationship between market quality index and distance to the nearest water point**


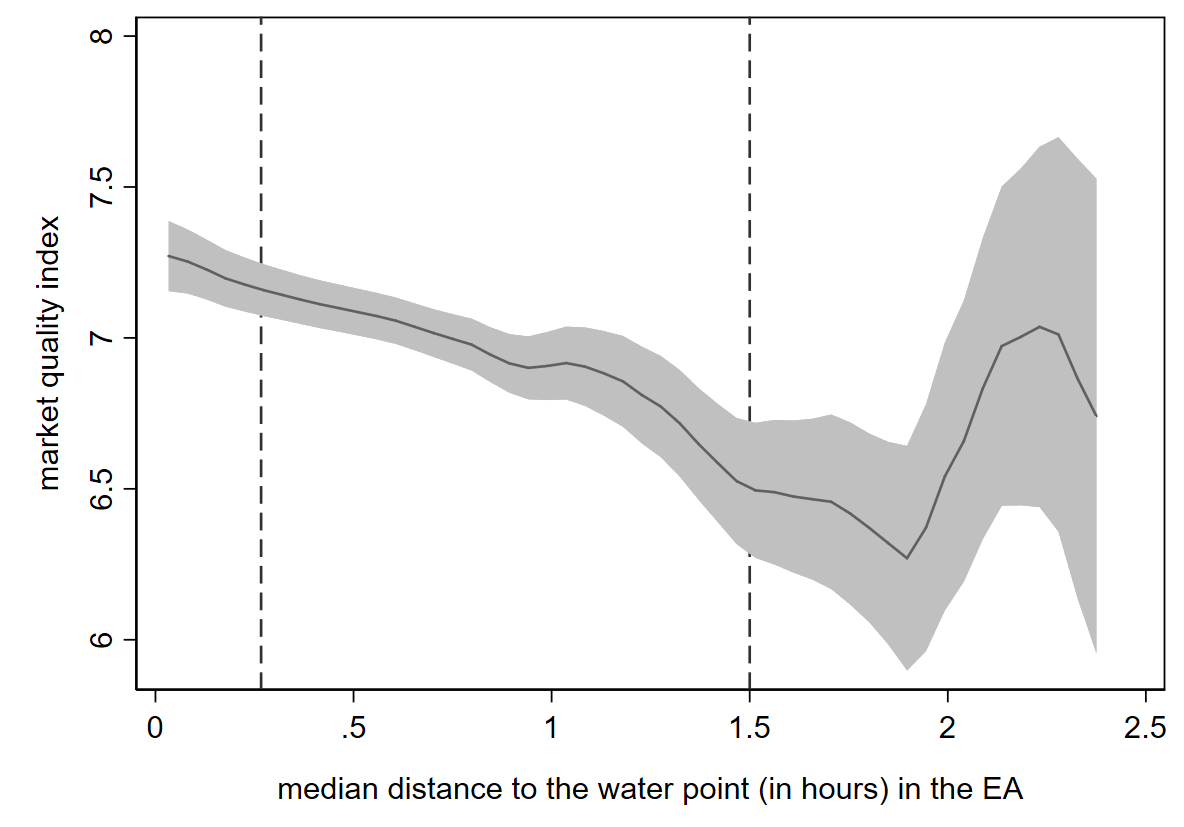


Note: Local polynomial regression. The shaded area represents 95%-confidence intervals. Vertical axis truncated at 2.75 hours; 99 percentile of the distance to the water point distribution. Dashed lines represent bottom and top 5% of the distance to the water point distribution.

**Figure S3: Relationship between market distance and distance to the nearest water point**


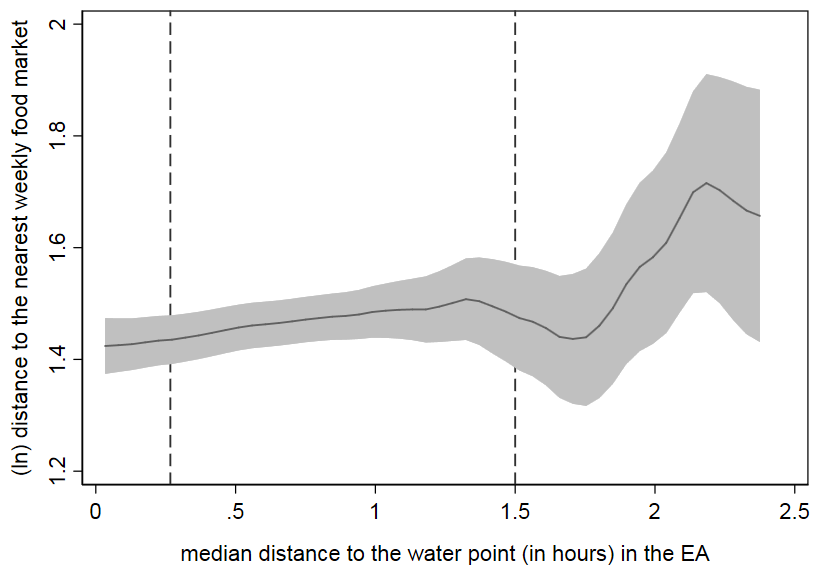


Note: Local polynomial regression. The shaded area represents 95%-confidence intervals. Vertical axis truncated at 2.38 hours; 99 percentile of the distance to the water point distribution. Dashed lines represent bottom and top 5% of the distance to the water point distribution.
